# Familial longevity is characterized by high circadian rhythmicity of serum cholesterol in healthy elderly individuals

**DOI:** 10.1111/acel.12547

**Published:** 2016-11-19

**Authors:** Rosa van den Berg, Raymond Noordam, Sander Kooijman, Steffy W. M. Jansen, Abimbola A. Akintola, P. Eline Slagboom, Hanno Pijl, Patrick C. N. Rensen, Nienke R. Biermasz, Diana van Heemst

**Affiliations:** ^1^ Department of MedicineDivision of EndocrinologyLeiden University Medical Center; ^2^ Einthoven Laboratory for Experimental Vascular Medicine; ^3^ Department of Gerontology and Geriatrics; ^4^ Molecular Epidemiology SectionDepartment of Medical Statistics and BioinformaticsLeiden University Medical CenterLeidenThe Netherlands

**Keywords:** aging, biological clock, cholesterol, circadian rhythm, longevity

## Abstract

The biological clock, whose function deteriorates with increasing age, determines bodily circadian (i.e. 24h) rhythms, including that of cholesterol metabolism. Dampening of circadian rhythms has been associated with aging and disease. Therefore, we hypothesized that individuals with a familial predisposition for longevity have a higher amplitude circadian serum cholesterol concentration rhythm. The aim of this study was to investigate circadian rhythmicity of serum cholesterol concentrations in offspring of nonagenarian siblings and their partners. Offspring from nonagenarian siblings (*n* = 19), and their partners as controls (*n* = 18), were recruited from the Leiden Longevity Study. Participants (mean age 65 years) were studied in a controlled in‐hospital setting over a 24‐h period, receiving three isocaloric meals at 9:00 h, 12:00 h and 18:00 h. Lights were off between 23:00 h and 8:00 h. Serum total cholesterol (TC), HDL cholesterol (HDL‐C), non‐HDL‐C and triglycerides (TG) were determined every 30 min over a 24‐h period. Serum TC concentrations were higher during day than during night in offspring (5.2 vs. 4.7 mm,* P* < 0.001) and in controls (5.3 vs. 5.0 mm,* P* < 0.001). The difference in TC concentrations between day and night tended to be greater in offspring than in controls (0.5 vs. 0.3 mm,* P* = 0.109), reaching statistical significance in females (*P* = 0.045). Notably, the day–night serum differences in non‐HDL‐C were twofold greater in offspring than in controls (0.43 vs. 0.21 mm,* P* = 0.044) and most explicit in females (0.53 vs. 0.22, *P* = 0.078). We conclude that familial longevity is characterized by a high circadian rhythmicity of non‐HDL‐C in healthy elderly offspring from nonagenarian siblings.

## Introduction

During the past decades, life expectancy has increased substantially, resulting in a growing number of individuals with a high age (Oeppen & Vaupel, 2002). Aging has been associated with an increased risk of dyslipidaemia (Steinhagen‐Thiessen *et al*., 2008). Familial human longevity has been associated with a less atherogenic lipid profile, which includes a larger LDL particle size (Barzilai *et al*., 2003), and lower circulating concentrations of triglycerides (Vaarhorst *et al*., 2011). Furthermore, multiple polymorphisms associated with human longevity were identified in genes involved in lipid metabolism, including several apolipoproteins (e.g. *APOB* De Benedictis *et al*., 1997; *APOA1* Garasto *et al*., 2003; *APOC1* Garasto *et al*., 2003 and *APOE* Soerensen *et al*., 2013 which are involved in lipid transport), uncoupling protein‐1 (which mediates triglyceride lowering effects through mediating thermogenesis in brown adipose tissue Rose *et al*., 2011), adiponectin (an anti‐inflammatory adipokine that increases β‐oxidation and triglyceride clearance Roszkowska‐Gancarz *et al*., 2012) and cholesteryl ester transfer protein (involved in determining the lipoprotein balance by mediating transfer of cholesteryl esters from HDL to LDL Barzilai *et al*., 2003; Soerensen *et al*., 2013).

Aging and lipid metabolism have both been related to functioning of the biological clock (Froy, 2011). The mammalian biological clock is a hierarchical system with a central clock, located in the suprachiasmatic nuclei (SCN) of the hypothalamus, and peripheral clocks at the tissue level (Reppert & Weaver, 2002). The SCN conveys circadian timing signals, such as light information, to peripheral clocks in the body through neuronal and hormonal cues (Reppert & Weaver, 2002). The biological clock ultimately serves to maintain circadian (i.e. 24 h) rhythms in bodily functions, such as sleep–wake cycles, hormone levels and plasma metabolites. In fact, human plasma lipid concentrations, including those of triglycerides and cholesterol, are rhythmic over a 24‐h period (Rivera‐Coll *et al*., 1994), independent of feeding and waking conditions (Chua *et al*., 2013).

The capacity of the biological clock to sustain circadian rhythms deteriorates with increasing age, which is reflected by reduced size of the SCN in the elderly, lower melatonin secretion and lower amplitudes of temperature rhythms (Hofman & Swaab, 2006). Animal studies indicate a crucial role for high circadian rhythmicity, that is high‐amplitude rhythms, in maintenance of health. For example, transplantation of SCN grafts from young to old animals restores the amplitude of electrical activity in the SCN and prolongs the lifespan of aged hamsters (Hurd & Ralph, 1998). Conversely, a lower amplitude of circadian rhythmicity in blood pressure has been associated with higher cardiovascular risk (White, 2000), independent of hypertension (Hermida *et al*., 2013). For this reason, we hypothesized that human familial longevity, which is associated with a lower risk of metabolic syndrome (Rozing *et al*., 2010) and cardiovascular and cardiometabolic diseases at advanced middle age (Westendorp *et al*., 2009), is associated with a higher rhythmicity in serum cholesterol concentrations. We investigated this hypothesis by comparing circadian rhythmicity of serum cholesterol concentrations between individuals with a familial predisposition for longevity and age‐matched controls.

## Results

### Characteristics of the study population

**Figure 1:**
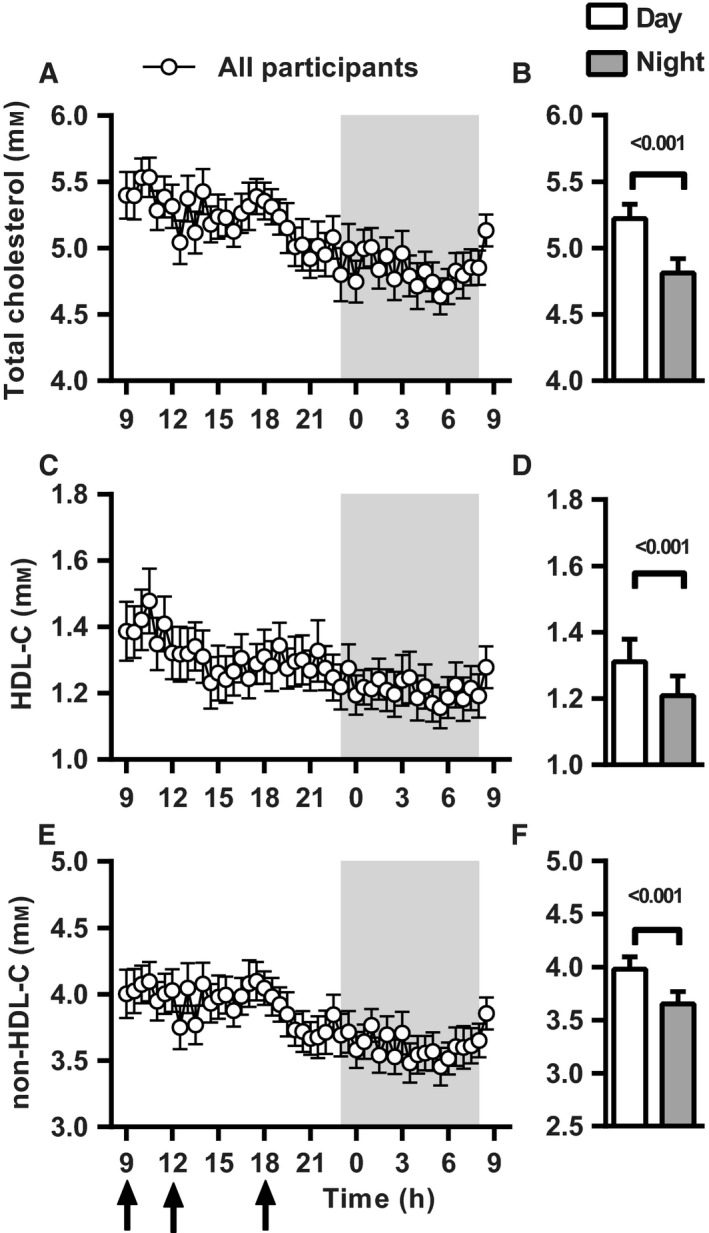
Circadian pattern of serum cholesterol concentrations in all participants combined. Mean serum cholesterol concentrations are displayed every 30 min over a 24‐h period for all participants combined (*n* = 37); total cholesterol (TC) (A), HDL cholesterol (HDL‐C) (C) and non‐HDL cholesterol (non‐HDL‐C) (E) Shaded area indicates dark/sleeping period. Black arrows indicate the time of three isocaloric meals (9:00 h, 12:00 h and 18:00 h). B, D and F present the mean ± SEM serum cholesterol concentrations during the day and night period for TC, HDL‐C and non‐HDL‐C, respectively.

We performed 24‐h venous blood sampling in 37 participants under highly controlled conditions at our research facility. During the 24‐h study period, participants minimized physical activity, received three isocaloric meals (at 9.00 h, 12.00 h and 18.00 h) and were allowed to sleep from 23.00 h to 8.00 h. The total study population consisting of 19 offspring from nonagenarian siblings (9 men, 10 women) and 18 of their partners as controls (10 men, 8 women), with a mean age of 65.0 ± 1.2 vs. 64.7 ± 1.2 years, respectively. The characteristics of the study population were similar between offspring and controls (Table 1), except that the mother's age at death or at inclusion of the study participants was higher in the offspring than in the controls (92.1 ± 1.8 vs. 78.6 ± 3.3 years, *P* < 0.01). Habitual sleep quality and chronotype were similar between offspring and controls (Table 1). We first characterized circadian rhythms in cholesterol concentrations for the entire study population. Serum total cholesterol (TC) and high‐density lipoprotein cholesterol (HDL‐C) concentrations were measured in samples collected every 30 min over the 24‐h period and from these measurements corresponding concentrations of non‐HDL‐C were derived. Twenty‐four hour mean concentration profiles for TC, HDL‐C and non‐HDL are shown in Fig. 1 together with the overall mean TC, HDL‐C and non‐HDL‐C concentration during the day period and night period. In the entire study population, serum concentrations of TC, HDL‐C and non‐HDL‐C exhibited an intra‐individual coefficient of variation (CV) of 13%, 19% and 14%, respectively. The mean concentrations of TC, HDL‐C and non‐HDL‐C were higher during the day than during the night (*P* < 0.001).

**Table 1:** Characteristics of the study population

	Offspring (*n* = 19)	Controls (*n* = 18)	*P*‐value
Men, *n* (%)	9 (47%)	10 (56%)	0.75
Age, years	65.3 ± 1.2	64.7 ± 1.2	0.71
Men	67.0 ± 2.2	64.9 ± 1.3	0.42
Women	63.9 ± 1.1	64.5 ± 2.1	0.78
BMI, kg/m^2^	24.5 ± 0.9	25.7 ± 1.0	0.32
Men	25.0 ± 1.1	25.9 ± 1.0	0.57
Women	24.0 ± 1.3	25.5 ± 1.9	0.50
Fat mass, %	31.8 ± 1.9	31.2 ± 2.0	0.83
Men	24.7 ± 1.1	25.0 ± 1.0	0.89
Women	38.3 ± 1.6	38.2 ± 2.0	0.99
Age father, years	82.3 ± 4.5	76.8 ± 2.2	0.28
Men	80 ± 8.2	76 ± 2.9	0.66
Women	85 ± 4.6	78 ± 3.4	0.27
Age mother, years	92.1 ± 1.8	78.6 ± 3.3	<0.01
Men	93 ± 2.6	82 ± 2.2	0.01
Women	92 ± 2.7	75 ± 6.9	0.03
Chronotype >0 min disturbance, *N* (%)^a^	5 (26.3)	4 (22.2)	0.77
Men	4 (44.4)	2 (20.0)	0.25
Women	1 (10.0)	2 (25.0)	0.40
Chronotype >30 min disturbance, *N* (%)	3 (15.8)	2 (11.1)	0.68
Men	3 (33.3)	1 (10.0)	0.21
Women	0 (0.0)	1 (12.5)	0.25
Sleep Quality PSQI, median (IQR)^b^	4.0 (2.0–7.0)	3.0 (1.0–5.0)	0.91
Men	3.0 (1.0–4.0)	2.0 (1.0–5.0)	0.78
Women	6.0 (4.0–9.0)	4.0 (1.0–6.0)	0.15

BMI, body mass index.

Data are presented as the mean ± SEM, unless indicated otherwise. BMI and fat mass were missing for 1 participant. *P*‐value calculated for offspring vs. controls, Student's *t*‐test.

a Scores obtained from Munich Chronotype Questionnaire (*P*‐values calculated with Mann–Whitney test).

b Scores obtained from Pittsburg sleep quality index, ranging from 0 (good sleep) to 21 (worse sleep; *P*‐values calculated with Mann–Whitney test).

### Comparison of circadian rhythmicity in serum cholesterol between offspring and controls

**Figure 2:**
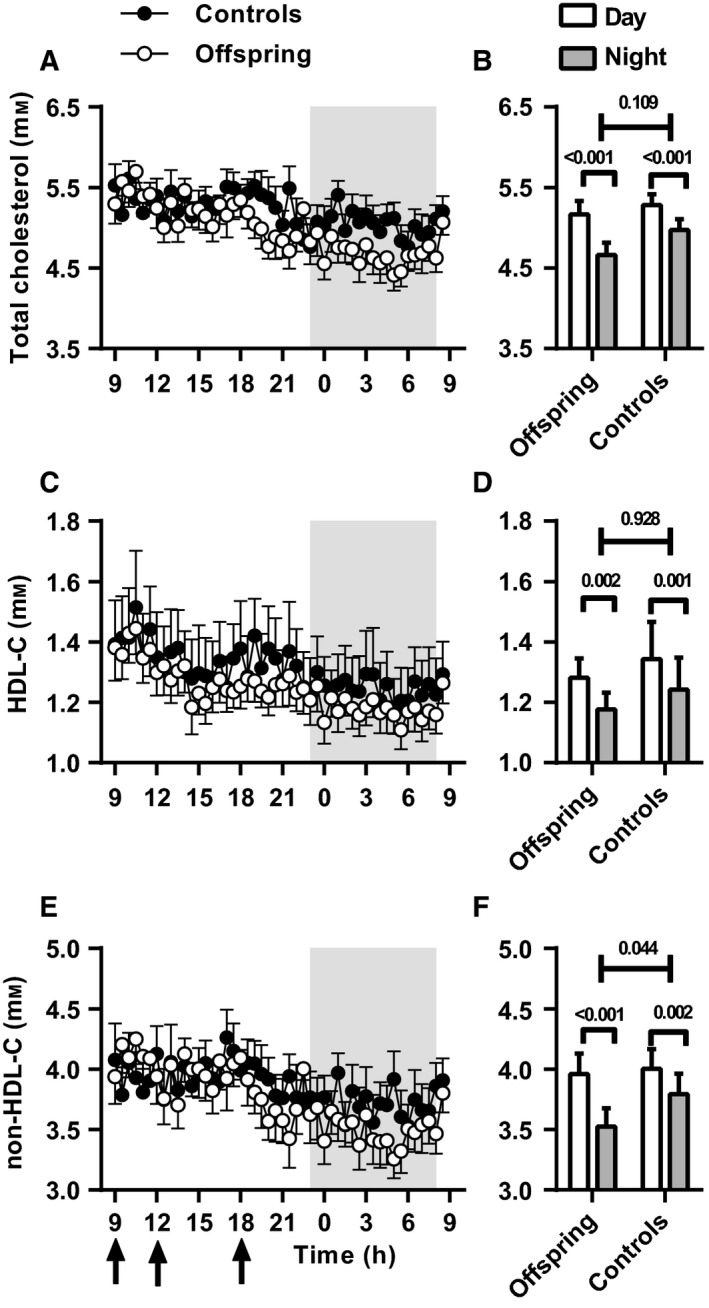
Serum cholesterol concentrations in offspring and controls. Mean serum cholesterol concentrations are displayed every 30 min over a 24‐h period stratified for offspring (*n* = 19; open circles) and controls (*n* = 18; solid circles); total cholesterol (TC) (A), HDL cholesterol (HDL‐C) (C) and non‐HDL cholesterol (non‐HDL‐C) (E). Shaded area indicates dark/sleeping period. Black arrows indicate the time of three isocaloric meals (9:00 h, 12:00 h and 18:00 h). B, D and F present the mean ± SEM serum cholesterol concentrations during the day and night period for TC, HDL‐C and non‐HDL‐C, respectively.

Mean serum concentrations of TC, HDL‐C and non‐HDL‐C are presented for every 30 min for 24 h stratified for offspring and controls in Fig. 2 together with the overall mean TC, HDL‐C and non‐HDL‐C concentration during the day period and night period. We observed no difference between offspring and controls in the 24‐h mean TC (*P* = 0.36), HDL‐C (*P* = 0.63) and non‐HDL‐C (*P* = 0.59; Table 2). The serum cholesterol concentrations as measured during the first and last time point of the 24‐h period were not statistically significantly different (data not shown). Both offspring and controls had higher serum concentrations of TC during the day than during the night (offspring: 5.2 ± 0.2 vs. 4.7 ± 0.2 mm;* P* < 0.001; controls: 5.3 ± 0.1 vs. 5.0 ± 0.1 mm;* P* < 0.001; Fig. 2A,B; Table 3). However, the day–night difference in TC concentrations tended to be larger in offspring than in controls (0.51 ± 0.10 vs. 0.31 ± 0.07 mm,* P* = 0.109). Mean HDL‐C concentrations were also higher during the day than during the night in both offspring (1.3 ± 0.1 vs. 1.2 ± 0.1 mm,* P* = 0.002) and controls (1.3 ± 0.1 vs. 1.2 ± 0.1 mm,* P* = 0.001; Fig. 2C,D; Table 3). However, the day–night difference in mean HDL‐C was similar between offspring and controls (*P* = 0.928). In line with the TC concentrations, the mean non‐HDL‐C concentrations were also higher during the day than during the night, both in offspring (4.0 ± 0.2 vs. 3.5 ± 0.2 mm,* P* < 0.001) and controls (4.0 ± 0.2 vs. 3.8 ± 0.2 mm,* P* = 0.002). Notably, the day–night difference in mean non‐HDL‐C concentrations was significantly larger in offspring than in controls (0.43 ± 0.09 vs. 0.21 ± 0.06 mm,* P* = 0.044; Fig. 2E,F; Table 3). As the non‐HDL lipoproteins transport triglycerides (TG) in addition to cholesterol, we determined TG concentrations. Similar to the HDL‐C concentrations, TG concentrations showed a difference between day and night, but this day–night difference was not different between offspring and controls (*P* = 0.852; Fig. S1A,B).

**Table 2:** Mean 24‐h serum cholesterol concentrations

	Offspring (*n* = 19)	Controls (*n* = 18)	*P*‐value
Total cholesterol (mm)	5.0 ± 0.2	5.2 ± 0.1	0.36
Men	4.9 ± 0.3	5.1 ± 0.2	0.58
Women	5.1 ± 0.2	5.3 ± 0.2	0.38
HDL‐C (mm)	1.2 ± 0.1	1.3 ± 0.1	0.63
Men	1.2 ± 0.1	1.1 ± 0.1	0.23
Women	1.3 ± 0.1	1.6 ± 0.2	0.14
Non‐HDL‐C (mm)	3.7 ± 0.2	3.9 ± 0.2	0.59
Men	3.7 ± 0.3	4.0 ± 0.2	0.41
Women	3.8 ± 0.2	3.7 ± 0.2	0.81

HDL‐C, HDL cholesterol; non‐HDL‐C, non‐HDL cholesterol.

Mean 24‐h parameters were calculated using the individual 24‐h serum cholesterol concentrations. Data are presented as means ± SEM. *P*‐value calculated for offspring vs. controls, Student's *t*‐test.

**Table 3:** Mean serum cholesterol concentrations during day and night

	Offspring (*n* = 19)		*P*‐value^a^	Controls (*n* = 18)		*P*‐value^a^	Offspring (*n* = 19)	Controls (*n* = 18)	*P*‐value^b^
	Day	Night		Day	Night		Day–Night difference	Day–Night difference	
Total cholesterol (mm)	5.2 ± 0.2	4.7 ± 0.2	<0.001	5.3 ± 0.1	5.0 ± 0.1	<0.001	0.51 ± 0.10	0.31 ± 0.07	0.109
Men	5.0 ± 0.3	4.7 ± 0.3	0.062	5.1 ± 0.2	4.9 ± 0.2	0.023	0.31 ± 0.14	0.27 ± 0.10	0.822
Women	5.3 ± 0.2	4.6 ± 0.2	<0.001	5.5 ± 0.2	5.1 ± 0.2	0.004	0.68 ± 0.11	0.36 ± 0.09	0.045
HDL‐C (mm)	1.3 ± 0.1	1.2 ± 0.1	0.002	1.3 ± 0.1	1.2 ± 0.1	0.001	0.10 ± 0.03	0.10 ± 0.03	0.928
Men	1.2 ± 0.1	1.2 ± 0.1	0.332	1.1 ± 0.1	1.0 ± 0.1	0.135	0.05 ± 0.04	0.05 ± 0.03	0.918
Women	1.4 ± 0.1	1.2 ± 0.1	<0.001	1.7 ± 0.2	1.5 ± 0.2	0.003	0.16 ± 0.03	0.16 ± 0.04	0.905
Non‐HDL‐C (mm)	4.0 ± 0.2	3.5 ± 0.2	<0.001	4.0 ± 0.2	3.8 ± 0.2	0.002	0.43 ± 0.09	0.21 ± 0.06	0.044
Men	3.9 ± 0.3	3.6 ± 0.3	0.025	4.1 ± 0.2	3.9 ± 0.2	0.038	0.33 ± 0.12	0.20 ± 0.08	0.388
Women	4.0 ± 0.2	3.5 ± 0.2	0.003	3.8 ± 0.2	3.6 ± 0.2	0.026	0.53 ± 0.13	0.22 ± 0.08	0.078

HDL‐C, HDL cholesterol; non‐HDL‐C, non‐HDL cholesterol.

Day (9:00 h – 23:00 h and 8.00 h – 9.00 h) and night (23:00 h – 8:00 h) mean values were calculated using the individual 24‐h serum cholesterol concentrations. Day–Night difference was calculated by subtracting the individual mean night value from mean day value. Data are presented as means ± SEM.

a *P*‐value calculated between day and night based on paired Student's *t*‐test.

b *P*‐value calculated of day–night difference between offspring and controls, Student's *t*‐test.

### Comparison in cholesterol rhythmicity between offspring and controls, stratified by sex

The analyses stratified by men and women are displayed in Table 3 and Fig. S1. Both in men and women, the mean serum TC concentrations during the day were higher than during the night (*P* < 0.05; Table 3; Fig. S1A–D). In both men and women, the day–night difference seemed higher in offspring than in controls; however, this was only statistically significant in women (0.68 ± 0.11 vs. 0.36 ± 0.09 mm,* P* = 0.045) and not in men (0.31 ± 0.14 vs. 0.27 ± 0.10 mm,* P* = 0.822). The HDL‐C concentrations in women were higher during the day than during the night in both offspring and controls (*P* < 0.05; Table 3; Fig. S1E,F), while this was not observed in men (*P* > 0.05; Table 3; Fig. S1G,H). In line with the observations in all subjects combined, both in men and women, there was no difference between offspring and controls in the day–night difference in HDL‐C concentrations. The mean non‐HDL‐C concentrations were higher during the day than during the night in both men and women (*P* < 0.05; Table 3; Fig. S1I–L). In women, the day–night differences in non‐HDL‐C tended to be larger in offspring than in controls (0.53 ± 0.13 vs. 0.22 ± 0.08 mm;* P* = 0.078; Table 3). Similarly, day–night differences in non‐HDL‐C concentrations in men were also slightly larger in offspring than in controls, but this was not statistically significant (0.33 ± 0.12 vs. 0.20 ± 0.08 mm;* P* = 0.388; Table 3).

## Discussion

The present study aimed to investigate differences in circadian rhythmicity of serum cholesterol concentrations between individuals with and without a familial predisposition for longevity. Irrespective of the study groups, we observed that serum cholesterol concentrations displayed a 24‐h rhythm with mean concentrations being higher during the day compared to the night. Previous studies on the daily variation in serum cholesterol concentrations have been conflicting. Some studies reported no variation during the day (Mirani‐Oostdijk *et al*., 1983; Persson *et al*., 2010), while others reported circadian variation in serum cholesterol with a CV up to 4% (Rivera‐Coll *et al*., 1994) and circadian variation in cholesterol precursors (Miettinen, 1982) or cholesterol production rate (Jones & Schoeller, 1990). However, in all these studies, sampling frequency was at most every 1.5 h and the study populations included up to 25 young adults, and the studies showing no circadian rhythm in cholesterol included only five individuals. Within the current study, sampling rate was every 30 min in 37 individuals, which therefore gives more detailed data about possible rhythmicity. Within our study population of 37 healthy older participants, we found a 24‐h variation in serum TC concentrations with a CV of 13%. Moreover, we observed a clear difference between day and night serum total cholesterol concentrations, showing that in this population of healthy elderly, cholesterol concentrations are rhythmic.

We observed that participants with a predisposition for familial longevity had a higher circadian rhythmicity (observed by a greater day–night difference) in cholesterol concentrations compared with their partners as controls. Aging has been associated with a decreased functioning of the biological clock, with respect to hormonal rhythms, core body temperature and sleep–wake cycles (Hofman & Swaab, 2006). Likely, the age‐related changes originate within the central biological clock, the SCN. Postmortem analysis showed that in individuals over 50 years of age, the SCN has decreased neuronal activity and rhythmicity compared to the SCN of younger individuals, as determined by arginine vasopressin expression (Hofman *et al*., 1996). Notably, the number of active SCN neurons correlated with the amplitude in activity patterns in elderly (Wang *et al*., 2015). Previously, we observed within the Leiden Longevity Study cohort that familial longevity is associated with higher mean thyroid stimulating hormone (TSH) concentrations, in the absence of differences in free T3 or T4 serum concentrations (Jansen *et al*., 2015a). We also observed a stronger temporal association between TSH and free T3 in offspring compared to in controls, which is indicative of a stronger correlation between circadian TSH and free T3 rhythms (Jansen *et al*., 2015b). Based on the current study, we speculate that a familial predisposition of longevity is characterized by subtle changes in some rhythmic parameters rather than their 24‐h mean serum concentrations resulting from a preserved biological clock function.

In the complete Leiden Longevity Study cohort, no difference in unfasted HDL‐C concentrations between offspring and controls was observed (Vaarhorst *et al*., 2011), in contrast to observations of Barzilai *et al*. (2001) in offspring of Ashkenazi Jewish centenarians and controls. In line with the larger Leiden Longevity Study cohort, in the present subset, no difference in HDL‐C rhythms between offspring and controls was found. The differences observed in the current study between offspring and controls were specifically in non‐HDL‐C concentrations during day and night, independent of triglyceride concentrations, habitual lifestyle and food intake. Therefore, there is likely an intrinsic difference in non‐HDL‐C metabolism in individuals with familial longevity.

The higher rhythmicity in non‐HDL‐C concentrations in offspring compared to controls could be a marker of a maintained clock function at a higher age. Alternatively, the increased rhythmicity in serum non‐HDL‐C concentrations could directly contribute to the increased life expectancy that is associated with familial longevity. The differences between the offspring and control group were mainly observed in non‐HDL‐C concentrations. This is particularly interesting with respect to life expectancy as non‐HDL‐C concentrations are implicated as causal in the pathogenesis of cardiovascular disease, which represents the number one cause of death worldwide. In humans, non‐HDL‐C concentrations mainly represent LDL‐C (Graham *et al*., 2012). LDL can be modified and taken up by macrophages, triggering atherosclerotic lesion development (Graham *et al*., 2012). Indeed, Mendelian randomization studies have demonstrated that lifelong low‐range LDL‐C concentrations (compared to higher LDL‐C concentrations) decreases cardiovascular risk (Postmus *et al*., 2015). Accordingly, long‐term high‐range cholesterol concentrations are associated with a significantly increased cardiovascular risk (Pletcher *et al*., 2010). It is therefore tempting to speculate that a stronger day–night rhythm of non‐HDL‐C (likely LDL‐C) contributes to a lower cardiovascular disease risk.

We investigated the sex‐specific effects on cholesterol rhythms in this study. Both men and women tended to display a higher rhythmicity in offspring than in controls in TC and non‐HDL‐C concentrations, but not in HDL‐C concentrations. However, this day–night difference was more prominent in women than in men, where the differences between offspring and controls did not reach significance, while in women, day–night difference in TC concentrations was significantly different and the day–night difference in non‐HDL‐C concentrations showed a trend. Of note, there is evidence pointing towards sex‐specific differences with respect to activity of the SCN. The SCN is sensitive to regulation by gonadal hormones (Bailey & Silver, 2014). Furthermore, in mice, aging‐induced decrease of the locomotor activity is higher in male mice than in female mice (Stowie & Glass, 2015). These data imply that also aging may affect biological clock function in a sex‐specific manner. This observation may also account for any possible sex differences in cholesterol rhythmicity in our study.

In this study, we observed that familial longevity is characterized by larger difference between night and day serum non‐HDL‐C concentrations. For human metabolism, the transition from night to day indicates the switch from fasting to feeding and thus from a catabolic to an anabolic state. This is, for example, apparent by the morning increase in insulin sensitivity (Yoshino *et al*., 2014). As cholesterol is a major substrate for synthesis of membranes and hormones, the observed increase of non‐HDL‐C upon waking may be a function of this anabolic state. The lower night concentrations of non‐HDL‐C are analogous to the diurnal rhythm of blood pressure. The absence of a decrease in blood pressure during the night, a phenomenon which is called ‘nondipping’, has been associated with increased cardiovascular risk (White, 2000), independent of hypertension (Hermida *et al*., 2013). However, as these studies were carried out in observational settings, the question remains whether the lower amplitude in blood pressure is a marker of underlying disease or a causal factor affecting cardiovascular disease risk. Animal studies support a causal role for dampened rhythms in metabolic disease. We previously showed that constant light exposure induces a dampened rhythm in SCN electrical activity as well as feeding and locomotor rhythms (Coomans *et al*., 2013). Interestingly, constant light increased weight gain independent of total food intake and activity, but by decreasing energy expenditure by decreasing brown adipose tissue activity (Kooijman *et al*., 2015).

A limitation of the current study is the relatively small sample size. Although our study cohort was larger than previous cohorts that studied 24‐h cholesterol rhythms, our sample size of 37 individuals is likely too limited to find subtle differences. This was also apparent from the stratification by sex. Visually, the HDL‐C concentrations in female control individuals seemed higher than those of female offspring; however, only two of the eight female control individuals displayed elevated 24‐h HDL‐C concentrations. Likewise, the differences in day–night non‐HDL‐C serum concentrations were larger in offspring than in controls, but only significant in women. Possibly, effects are more subtle in men, which were therefore not detected in a small study such as the present one.

In conclusion, we show that in advanced middle age, familial longevity is characterized by a high rhythmicity of serum non‐HDL‐C concentrations in a subset of healthy individuals. Our study supports the hypothesis that high‐amplitude rhythms may contribute to healthy aging and therefore decreased rhythmicity may be an additional risk factor for development of disease. Increasing circadian rhythmicity may be an attractive target to promote longevity.

## Experimental procedures

### Ethics statement

The Medical Ethical Committee of Leiden University Medical Center approved this study. The study was performed according to the Helsinki Declaration. Written informed consent was obtained from all study participants.

### Study participants

Participants were recruited from the Leiden Longevity Study, which aims to investigate genetic factors and biomarkers associated with familial longevity. A more detailed description of the study design and recruitment strategy of the Leiden Longevity Study has been described elsewhere (Schoenmaker *et al*., 2006). In short, a total of 421 long‐lived families were recruited, without selection based on health condition or demographics. Families were included when at least two long‐lived siblings were still alive and fulfilled the age criteria of 89 years for men and 91 years for women. In total, 1671 offspring of these long‐lived individuals were recruited and 744 partners thereof as controls.

Of these, a subsample of 38 healthy participants were recruited in the Switchbox study for in‐ depth endocrine and metabolic phenotyping (Jansen *et al*., 2015a), which included 24‐h venous blood sampling. To be included in the Switchbox study, participants had to have a fasting glucose level <7 mm, haemoglobin level <7.1 mm, a body mass index (BMI) between 19 and 33 kg/m^2^ and had to be free of any significant chronic disease. These conditions included, among others, renal, hepatic or endocrine diseases as well as the use of medication known to influence lipid concentrations or interfere with hormonal axes. A complete list of criteria that were considered prior to study inclusion has been published elsewhere (Akintola *et al*., 2015). Of the 38 participants, one participant was excluded because of a newly diagnosed hypertriglyceridemia, leaving 37 participants for the present study (19 offspring and 18 controls). Habitual sleep quality was assessed using the Pittsburgh sleep quality index (PSQI) questionnaire (Buysse *et al*., 1989; Carpenter & Andrykowski, 1998). With this questionnaire, different aspects of sleep are subjectively assessed (e.g. sleep onset latency, sleep quality and daytime dysfunction). A summary score was calculated to obtain an overall impression of a person's quality of sleep, ranging from a score of 0 (good sleep) to 21 (worse sleep). Chronotype was assessed using the Munich Chronotype Questionnaire, which has been described and validated before (Roenneberg *et al*., 2003; Zavada *et al*., 2005). For the present study, we assessed whether a person has any shortage of sleep during a normal week day.

### Study and sampling procedure

After an overnight fasting of 10–14 h, a catheter was inserted before start of the study, and blood sampling started at 09:00 h. During 24 h, every 10 min 1.2 and 2 ml blood was collected in a K_3_‐EDTA tube and a serum separator (SST) tube, respectively. Participants received three standardized meals at three fixed time points (namely between 09:00 h‐10:00 h, 12:00 h‐13:00 h and 18:00 h‐19:00 h). All meals consisted of 600 kcal Nutridrink, containing a standard percentage of energy derived from fat (35%), carbohydrates (49%) and protein (16%; Nutricia Advanced Medical Nutrition, Zoetermeer, the Netherlands). All participants were sampled year‐round in the same room with standardized ambient conditions. Offspring and control couples were studied on the same day. Participants were not allowed to sleep during the day, and except for lavatory use, no physical activity was allowed during the study period; lights were turned off between 23:00 h to 08:00 h to allow the participants to sleep.

### Biochemical analyses

After blood withdrawal, the serum tubes were kept at room temperature and immediately centrifuged when the samples were clotted. Serum was aliquoted into 500‐μl tubes and stored at −80 °C. For the present study, we determined serum concentrations of total cholesterol (TC), high‐density lipoprotein cholesterol (HDL‐C), non‐HDL‐C and triglycerides (TG) every 30 min. TC and TG were analysed by a commercially available enzymatic kit according to the manufacturer's protocols (Roche, Mannheim, Germany), with an interassay coefficient of variation (CV) of 7% and intra‐assay CV of 3%. HDL was isolated by precipitation of ApoB‐containing lipoproteins, with an interassay CV of 13% and intra‐assay CV of 4%. Hereto, 20% polyethylene glycol (Sigma‐Aldrich) in 200 mm glycine‐buffered saline (pH 10) was added to serum, centrifuged for 30 min at 3.5 G, and HDL‐C was determined in the supernatant. Non‐HDL‐C was calculated by subtracting HDL‐C from TC.

### Anthropometrics

At the study centre, we measured the height, weight and body fat percentage of the participants. Weight (in kg) was divided by the squared height (in m) to calculate the body mass index (BMI). The percentage of body fat was measured using a bioelectrical impedance analysis (BIA) meter at a fixed frequency of 50 kHz (Bodystat^®^ 1500 Ltd, Isle of Man, British Isles).

### Statistical analyses

We calculated the overall mean TC, HDL‐C and non‐HDL‐C per participant for the whole 24‐h study period, for the day period (9.00 h – 23.00 h and 8.00 h – 9.00 h) and the night period (23.00 h – 8.00 h). Comparisons between offspring and controls were performed using independent sample Student's *t*‐tests (continuous outcomes) and with chi‐square statistics (dichotomous outcomes). We used paired sample Student's *t*‐tests to analyse differences between day and night serum cholesterol values. To assess whether the differences in mean TC, HDL‐C, non‐HDL‐C and TG between day and night were different between offspring and controls, we calculated the individual mean difference between mean day and night values and tested the differences using independent sample *t*‐tests. All analyses for the comparisons between offspring and controls were additionally stratified by sex. All statistical analyses were conducted using spss v.20 for Windows (SPPS Inc., Chicago, IL, USA). Data are presented as means ± SEM. Because analyses were not independent from each other, we did not correct for multiple comparisons. Therefore, a two‐sided *P*‐values below 0.05 were considered statistically significant.

## Author contributions

RvdB designed the study, supervised the cholesterol measurements, conducted the analyses and wrote the initial version of the manuscript. RN designed the study, conducted the analyses and wrote the initial version of the manuscript. SK designed the study and wrote the initial version of the manuscript. SWJ and AAA included the study participants and collected the biomaterials and data of the Switchbox Study. HP, PES, NRB, PCNR and DvH supervised the project. All authors critically commented on the initial versions of the manuscript and approved the final version of the manuscript for submission.

## Funding

PCN Rensen is an Established Investigator of the Dutch Heart Foundation (grant 2009T038). This study was supported by the Netherlands Organisation for Scientific Research (NWO‐VENI grant 016.136.125 to NR Biermasz) and funded by the European funded projects Switchbox (FP7, Health‐2010‐259772) and HUMAN (FP7, Health‐2013‐INNOVATION‐1‐602757).

## Conflict of interest

The authors have nothing to disclose.

## Supporting information


**Fig. S1** Serum triglyceride concentrations in offspring and controls.
**Fig. S2** Serum cholesterol concentrations in offspring and controls in women and men.
