# Supplementary material for: Familial longevity is characterized by high circadian rhythmicity of serum cholesterol in healthy elderly individuals
Source: Aging Cell. 2016 Nov 19;16(2):237–43. doi: 10.1111/acel.12547 (PMC5334529; doi:10.1111/acel.12547)
Supplement: Supplementary file 1 — Fig. S1 Serum triglyceride concentrations in offspring and controls. Fig. S2 Serum cholesterol concentrations in offspring and controls in women and men. [file ACEL-16-237-s001.pdf]

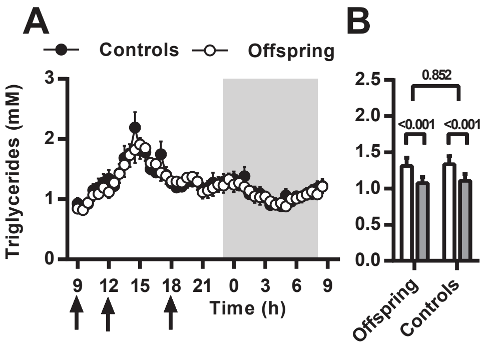

Supplemental Figure 1: Serum triglyceride concentrations in offspring and controls
Mean serum triglycerides (TG) concentrations are displayed every 30 min over a 24h period stratified for offspring (n=19) (open circles) and controls (n=18) (solid circles) (**A**), the mean ± SEM serum TG concentrations during the day and night period (**B**). Shaded area indicates dark/sleeping period. Black arrows indicate the time of three isocaloric meals (9:00h, 12:00h, and 18:00h).

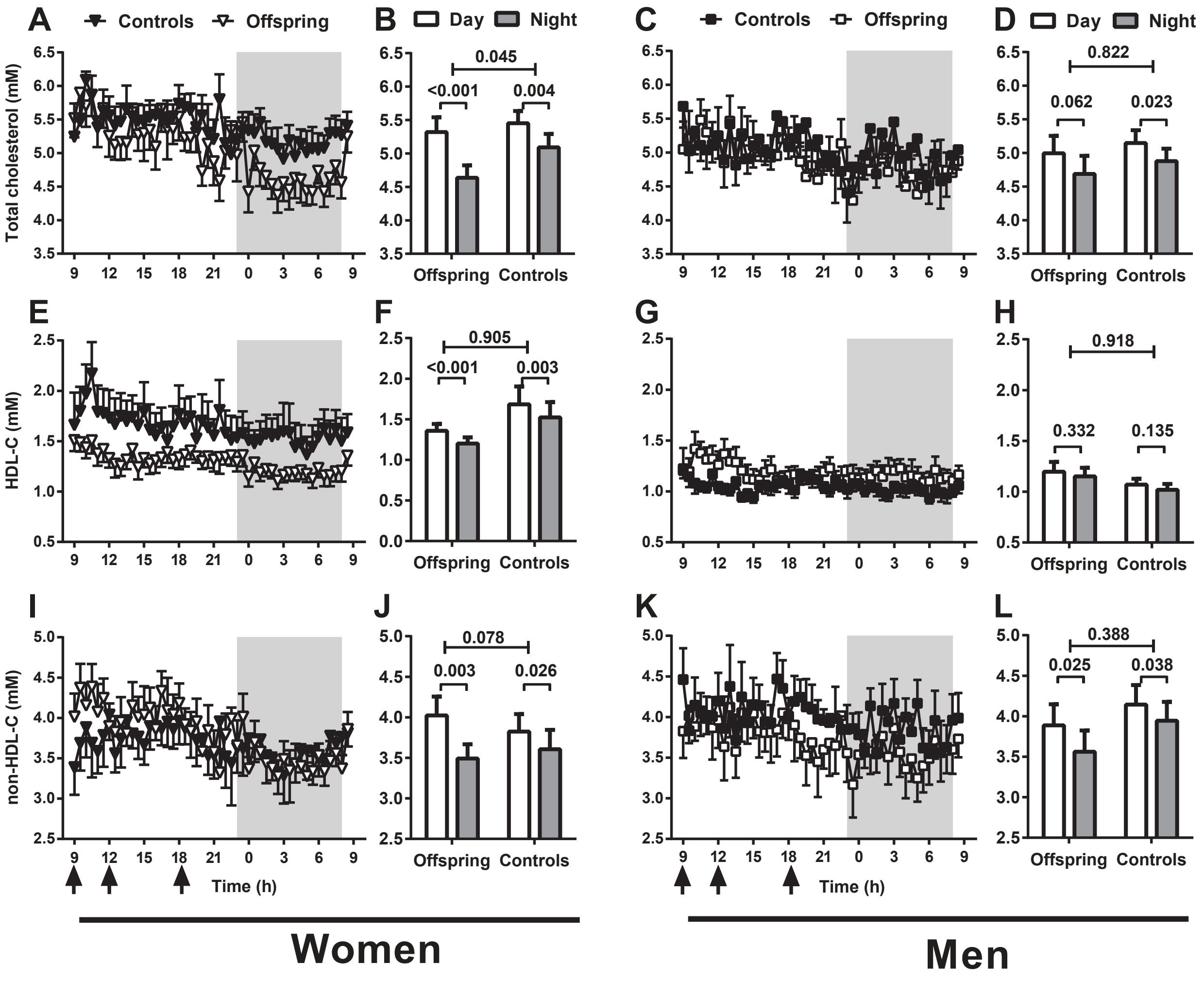

Supplemental Figure 2: Serum cholesterol concentrations in offspring and controls in women and men
Mean serum cholesterol concentrations are displayed every 30 min over a 24h period separately for women (triangles) and men (squares), and stratified for offspring (open symbols) and controls (solid symbols); total cholesterol (TC) (**A;C**), HDL-cholesterol (HDL-C) (**E;G**), and non-HDL-cholesterol (non-HDL-C) (**I;K**). Shaded area indicates dark/sleeping period. Black arrows indicate the time of three isocaloric meals (9:00h, 12:00h, and 18:00h). Figures **B, D, F, H, J,** and **L** present the mean ± SEM serum cholesterol concentrations during the day and night period for TC (**B, D**), HDL-C (**F, H**), and non-HDL-C (**J, L**), respectively.
